# Ionic Liquids as Promisingly Multi-Functional Participants for Electrocatalyst of Water Splitting: A Review

**DOI:** 10.3390/molecules28073051

**Published:** 2023-03-29

**Authors:** Chenyun Zhang, Puyu Qu, Mei Zhou, Lidong Qian, Te Bai, Jianjiao Jin, Bingwei Xin

**Affiliations:** 1 School of Intelligent Manufacturing, Wuxi Vocational College of Science and Technology, Wuxi 214028, China; 2 College of Chemistry and Chemical Engineering, Dezhou University, Dezhou 253023, China

**Keywords:** ionic liquid-derived electrocatalyst, reactive reagent, water splitting, hydrogen evolution reaction, oxygen evolution reaction

## Abstract

Ionic liquids (ILs), as one of the most concerned functional materials in recent decades, have opened up active perspectives for electrocatalysis. In catalyst preparation, ILs act as characteristic active components besides media and templates. Compared with catalysts obtained using ordinary reagents, IL-derived catalysts have a special structure and catalytic performance due to the influence of IL’s special physicochemical properties and structures. This review mainly describes the use of ILs as modifiers and reaction reagents to prepare electrocatalysts for water splitting. The designability of ILs provides opportunities for the ingenious composition of cations or anions. ILs containing heteroatoms (N, O, S, P, etc.) and transition metal anion (FeCl_4_^−^, NiCl_3_^−^, etc.) can be used to directly prepare metal phosphides, sulfides, carbides and nitrides, and so forth. The special physicochemical properties and supramolecular structures of ILs can provide growth conditions for catalysts that are different from the normal media environment, inducing special structure and high performance. ILs as heteroatom sources are safe, green and easy to operate compared with traditional heteroatom sources. The strategy for using ILs as reagents is expected to realize 100% atomic transformation of reactants, in line with the concept of green chemistry. This review reflects the discovered work with the best findings from the literature. It will offer readers a deeper understanding on the development of IL-derived electrocatalysts and inspire them to ingeniously design high-performance electrocatalysts for water splitting.

## 1. Introduction

Ionic liquids (ILs) are a class of molten organic or inorganic salts with a melting point below 100 °C, preferably around room temperature [1,2,3]. They are composed of cations and anions. Their common cations include imidazolium ions, pyrrole ions, quaternary ammonium ions, quaternary phosphonium ions, sulfonium, pyrazolium, guanidinium, etc., while anions involve halogen ions, tetrafluoroborate ions, hexafluorophosphate ions, metallic anions, etc. The main reason that ILs present a low melting point is due to the asymmetry of anions and cations, which prevents ions from stacking regularly into crystals [4,5,6]. The convenient combination of cations with anions makes ILs “designer solvents”, inducing the rational and flexible design and synthesis of new ILs [7,8,9,10]. Therefore, the variety of ILs is increasing with the deepening of research. Compared to traditional organic solvents, ILs are considered green solvents due to their low vapor pressure and non-volatility, although more studies have found their toxicity [11]. For several decades, ILs as green solvents and functional materials have been a popular research topic in chemistry, biology, physics, engineering and many other fields [12,13]. In organic synthesis, the involvement of ILs in the formation of C−C, C−O, C−S, and C−N bonds has been investigated [14,15]. IL-assisted Mannich reaction, Michael addition and so forth have been found to be a promising synthetic trail [16,17,18]. Generally, organic reactions based on ILs react quickly and reduce side reactions. ILs act as not only reaction media but also catalysts in the formation of these covalent bonds. More importantly, IL media can be reused, which lowers environmental pollution by coordinating with their nonvolatility. In the field of electrochemistry, ILs are widely used due to their high ionic conductivity. They are considered safe electrolytes for various energy storage devices, such as batteries, solar cells, batteries, fuel cells, thermoelectric chemical cells, and supercapacitors [3]. In the preparation of inorganic catalysts, IL plays a variety of roles such as catalyst, solvent, and catalyst carrier. The special physical and chemical properties and liquid structure of ILs change the formation mechanism of catalysts, thereby regulating their catalytic performance.

ILs have special physicochemical properties and supramolecular structures that have fundamentally altered the concept of liquid. Therefore, ILs have multiple roles in the field of catalysis [13,19,20]. They are widely used as media and templates to offer high-performance catalysts because they can dissolve many substances that cannot be dissolved in water or organic solvents [20]. In recent years, the concept of fewer or even no emissions of chemicals has become a worldwide consensus. Therefore, using ILs as modifiers and reactants directly to prepare catalysts has become a research hotspot [21,22,23]. Researchers rationally and flexibly design the cations and anions species of ILs involving N, P, S, C and even metal elements to prepare catalysts [24]. To date, many kinds of IL-derived electrocatalysts, including transition metal-based catalysts, N-doped carbon materials, metal and heteroatom co-doped carbon materials, among others, have been synthesized. The supramolecular structures of ILs have inheritance characteristics; therefore, they can act as structure-directing agents to shape the morphologies and structures of catalysts through providing special solvent environment for their growth as well as modulating the synthesis mechanism [25,26]. The change of morphologies and mechanism of catalysts is expected to tailor the catalytic performance.

Various countries have attached great importance to the research of new energy technologies because of fossil energy crisis and environmental pollution. Hydrogen, as a new type of green energy with high energy density, has attracted worldwide attention [27,28]. In recent years, various hydrogen sources have been discovered, such as ethanol, urea, among others [29,30]. Electrocatalytic water splitting for hydrogen production has the advantages of being a simple process, high hydrogen purity and no carbon emissions [31]. The two half reactions in water splitting, hydrogen evolution reaction (HER) and oxygen evolution reaction (OER), are two-electron and four-electron reactions, respectively. The HER process mainly includes two steps (adsorption and desorption). The OER requires the breaking of O–H bonds and the formation of O–O bonds [32]. According to the Gibbs free energy, water splitting occurs at the theoretical voltage 1.23 V. However, the applied voltage in the actual process is much greater than 1.23 V because energy consumption generates during the adsorption and desorption. Therefore, it is necessary to explore efficient, powerful electrocatalysts to reduce the overpotential and additional electrical energy consumption of water electrolysis. Precious metal-based electrocatalysts, such as Pt, RuO_2_, and IrO_2_, have excellent properties [33,34]. However, their industrial production is hindered by low reserves and high prices. Therefore, there is an urgent need to develop cheap catalysts, such as transition-metal catalysts or carbon materials, etc. [35,36]. Transition-metal catalysts not only are cheap but they also have d electrons, which are convenient for the transmission of electrons in the process of catalytic water electrolysis. They are expected to replace noble metals for catalytic electrolysis of water, while carbon-based materials are abundant and simply functionalized to tailor performance. Researchers have been dedicated to developing strategies on how to improve the catalytic activity of these non-precious catalysts. It is found that the compositions, special physicochemical properties and supramolecular structure of ILs can be attributed to catalysts with excellent crystal phases, geometrical morphologies and properties, etc. Meanwhile, the advocacy of green chemistry inspires the exploration of environmentally friendly media and new synthetic raw materials. In this case, using ILs as participants to prepare electrocatalysts has received great attention [37,38].

In this paper, the achievement of ILs as either modifiers or reactive reagents to prepare electrocatalysts for water splitting is reviewed. The article is divided into four parts. The Introduction section includes the concept of ILs and the urgency of using them to prepare electrocatalysts for water splitting. In Section 2, ILs are described as modifiers to the electrocatalysts for water splitting. As modifiers, ILs can be modified on the surface of electrocatalysts to change their properties, such as the performance of transmission electron, hydrophobicity, conductivity, among others. Section 3 explores ILs as reactive reagents in the synthesis of catalysts based on carbon materials and transition metal compounds with many advantages. In Section 4, the conclusion about this review is offered. In addition, the existing problems and improvement prospects about ILs participating in the preparation of catalysts are further analyzed.

## 2. Ionic Liquids as Modifiers for HER and OER Electrocatalysts

Using ILs to modify the catalyst surface is one of the most important strategies in surface engineering [39]. Compared with ordinary surface-modifying material, ILs as modifiers can transfer the special physical and chemical properties to catalysts, such as conductivity, stability and other properties. ILs’ special properties and structure are expected to improve the electron and mass transport in the process of electrolytic water. Some IL-derived electrocatalysts accelerate the adsorption and desorption process to promote the HER reaction, while some IL-assisted catalysts favor -O-H breaking in OER. Both IL monomers and polymer ionic liquids (poly(IL)s) have been used to cover the catalyst surfaces through physical adsorption, coordination or covalent bonding, becoming part of the catalysts.

**Figure 1:**
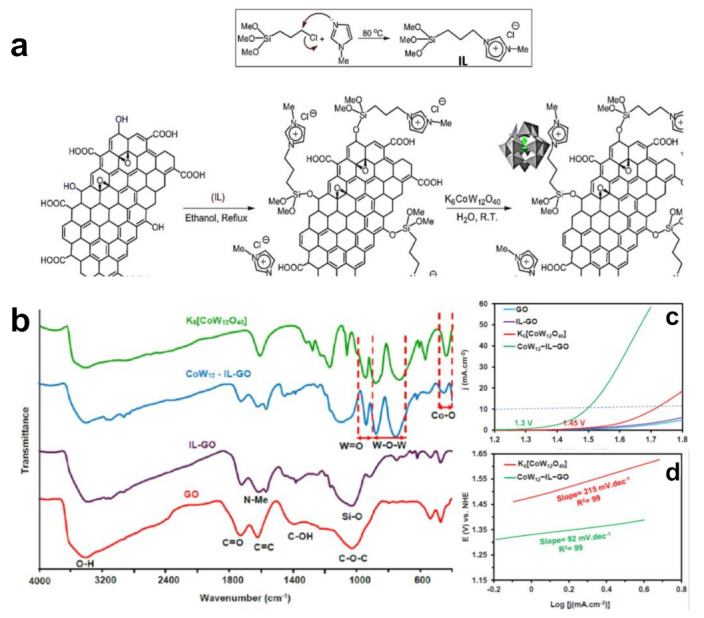
Schematic diagram of preparation of CoW_12_−IL−GO hybrid nanomaterials. (**a**) FT−IR spectra of CoW_12_−IL−GO and control samples; (**b**) OER polarization curves; (**c**) and Tafel slopes (**d**) of CoW_12_−IL−GO and control samples. Reprinted with permission from ref. [48], copyright 2022 Elsevier.

Taking the hybrid structure of [C2mim]TfO and (TiO_2_)_n_ nanoclusters as an example, Singh et al. systematically studied the effect of IL monomer on HER through first-principle calculations [40]. They found that IL monomer could theoretically improve the catalytic activity of catalysts. Li proved the correctness of the calculation results through experiments [41]. They functionalized in situ carbon nanotubes (CNT) catalysts through a series of imidazolium-based IL, denoted as CNTs−IM−X (X = Br^−^, Cl^−^, BF_4_^−^ and PF_6_^−^), and proved that CNT modified using ILs presented superior electrocatalytic performance for HER than unmodified CNT. Theoretical and experimental results show that the functionalization of CNT with imidazolium-based IL not only promotes the electron transfer process but also acts as the electron receptor with excellent hydrogen adsorption ability, benefiting the mass transfer during the water splitting process [42,43,44,45,46,47]. In addition, different anions of ILs lead to a different catalytic performance. CNT−IM−Cl required onset overpotential of 80 mV with the Tafel slope of 38 mV dec^−1^ for HER, better than CNTs−IM−X (X = Br^−^, BF_4_^−^ and PF_6_^−^) [41]. This result further indicates that IL monomers play regulatory role in the catalytic activity. Anions participate in the water electrolysis process through the interaction between anions and H^+^ or H_2_O, thus improving the electrolysis speed and yield. This characteristic provides an interesting route for improving catalyst performance. Cl^−^ in Cl-IL-GO was exchanged into Keggin type [CoW_12_O_40_]_6_^−^ polyanion (denoted as CoW_12_), as shown in Figure 1a,b [48]. As-prepared CoW_12_−IL−GO had a superior oxidation–reduction property and special stability under high oxidation conditions. It had a low overpotential of 269 mV at 10 mA·cm^−2^ with small Tafel slope (92 mV·dec^−1^) under neutral conditions (Figure 1c,d).

Poly(IL)s, combining the advantages of ILs with polymers, are generally polymerized by IL monomers containing alkenyl or alkynyl group [49], among others. Poly(IL)s have been extensively studied in the field of water splitting due to their excellent features [50]. In fact, poly(IL)s themselves are a kind of good electrocatalysts for the electrolysis of water [51]. Taking imidazolium-based poly(IL)s as examples [52], the existence of N atoms as electron-rich dopant in the imidazolium ring will induce high electron density. As a result, C2 appears positive charge density, favoring the adsorption of OH^−^ during catalyzing OER. Meanwhile, the poly(IL)s can form preferential water channels to aid the transport of protons between imidazolium rings and water molecules.

However, poly(IL)s are less conductive. It is often necessary to hybridize with conductive materials (e.g., carbon-based materials, metal nanoparticles, metal compounds, etc.) to improve their catalytic activity. The numerous advantages of poly(IL)s will endow these hybrids with designable structures, adjustable solubility, chemical and thermal stability, and so forth [39,52,53,54]. 

Poly(IL)-carbon material mixtures/hybrids can be prepared by mechanically mixing poly(IL) with carbon-based materials or covalently grafting ILs through functional groups (e.g., −NH_2_, −COOH, etc.) on substrates. These IL-modified carbon-based mixtures/hybrids exhibit promising HER performance due to their strong proton adsorption ability and conductivity.

**Figure 2:**
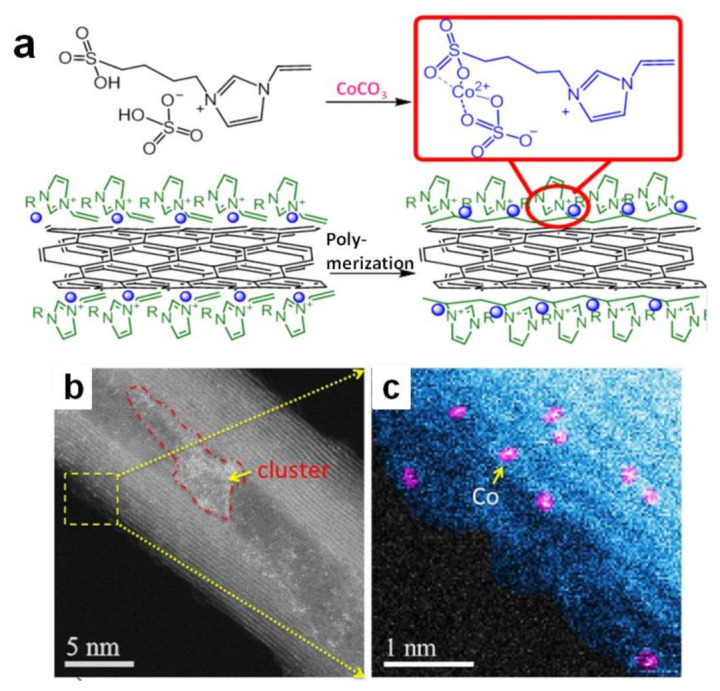
Schematic illustration for the fabrication process of Co-poly(IL)/CNT (**a**); ADF-STEM images (**b**); of this simple(**c**). Reprinted with permission from ref. [56], copyright 2018 Wiley.

Poly(IL)-metal hybrids are another kind of effective electrocatalysts for water splitting. Pt or Pd immobilized in situ on poly(1-vinyl-3-methylimidazolium) (poly(VIMM)) strongly boosts the catalytic performance for OER [52,55]. It is pleasing that the combination of non-noble metal catalysts with poly(IL) can also achieve good results [56,57,58,59]. A functional IL whose imidazolium cation involved sulfate was designed (Figure 2a) [56]. It could combine with Co^2+^ through a simple chemical reaction between sulfate and CoCO_3_. The complex containing IL and Co^2+^ was set on carbon nanotube surface via physisorption. The following thermal initiation free radical polymerization process offered Co-poly(IL)/CNT catalyst (Figure 2b,c), which the IL monomers transformed to poly(IL) while the metal nanoparticles were immobilized in situ on the poly(IL). Combining the metal ions with IL could not only lead metal nanoparticles to be uniformly distributed in poly(IL)s, but also adjust the electron structure of atomically dispersed Co. This catalyst was favorable for OER. Moreover, insolubility of poly(IL) species in water kept the counter Co^2+^ stable on CNT surfaces during the electrolysis process, exhibiting a good OER durability. Similarly, NiS_2_−MoS_2_ heterostructure was immobilized on polypyrrole/graphene oxide functionalized by poly(1-vinyl-3-ethylimidazolium bromide) (PVEIB) via an ordinary hydrothermal procedure, and then exhibited the excellent electrocatalytic performance for HER [59]. Similarly, poly(3-(1-vinylimidazolium-3-yl) propane-1-sulfonate) polypyrrole/graphene oxide (PVIPS/PPy/GO) was grown on MoS_2_−SnS_2_ heterogeneous nanoplate in situ, which could greatly improve the nitrogen reduction performance [60].

## 3. Ionic Liquids as Reactive Reagents for Electrocatalysts of HER and OER

Although ILs have good thermal stability, decomposition still occurs under appropriate conditions, such as at high temperatures [61,62,63]. The resulting elements stemmed from ILs are related to their components. Generally, cations of ILs contain generally C, N elements. The anion components of ILs are more flexible than cations, and can be ingeniously designed to involve N, S, P, etc., even metal anions (such as FeCl_4_^−^, NiCl_3_^−^, CoCl_3_^−^) [43,64,65,66,67]. ILs as agents to prepare catalysts have many advantages: (1) Compared with traditional heteroatom sources, ILs are safe, green and easy to operate. (2) ILs have special physicochemical properties and the supramolecular structures. This not only provides the growth conditions for catalysts that are different from the normal media environment, but also has the ability to shape the morphologies of nanomaterials, which can induce catalysts with special structure, and then tune their performance. (3) ILs as reagents are expected to realize 100% atomic transformation of reactants. In order to achieve the goal of carbon peak and carbon neutralization, it is necessary to reduce the discharge of solvent and improve the utilization rate. IL-derived catalysts show good catalytic performance in electrolytic water. Therefore, the direct use of ILs as reagents in the preparation of catalysts has received widespread attention. 

### 3.1. Ionic Liquids as Reactants to Prepare Heteroatom-Doped Carbon Materials as the Electro Catalysts for Water Splitting

Carbon-based materials (e.g., CNT, graphene, carbon nanofiber, etc.) are promising metal-free catalysts for HER as well as OER because of their particular properties of high surface area, long chemical durability and low cost [41,68]. However, their uniform charge distribution results in low catalytic activity. It is known that doping other atoms (such as N, S, P, etc.) into carbon materials can introduce more active sites and greatly tailor electronic structure and properties of adjacent C atoms via delocalizing electrons around carbon atoms, which is a valid strategy for improving electrocatalytic activity [69,70,71]. Especially, N-doped carbon (N−C) materials can effectively lower the Gibbs free energy of adsorption through inducing charge delocalization of C atoms [72]. Therefore, N-doped carbon materials have drawn great attention in the field of electrochemical catalysis.

It is found that carbonizing the combination of ILs or poly(IL)s with carbon materials is a common synthesis strategy to obtain N-, N,P-, N,S- even N,P,F-co-doped C-based materials, in which heteroatoms originate from ILs [73,74]. For example, carbonizing the mixture of *N,N*-bis(4-(methoxycarbonyl)benzyl)-*N*-methyl-*d*-glucaminium hexaflorophosphate ([MBMG]PF_6_) and graphene obtains N,P-graphene [75]. Similarly, carbonizing the mixture of poly(1-vinyl-3-cyanomethylimidazolium bis(trifluoromethylsulfonyl)imide ([PCMVIM]Tf_2_N) and bovine serum albumin forms N,S-co-doped micro/mesoporous C nanomaterials [76]. Their catalytic activity and durability are related to calcination temperature. The resulting product pyrolyzed at 1000 °C presents ƞ_10_ = 172 mV for HER in acid solution, as well as ƞ_10_ = 460 mV for OER in alkaline solution.

**Figure 3:**
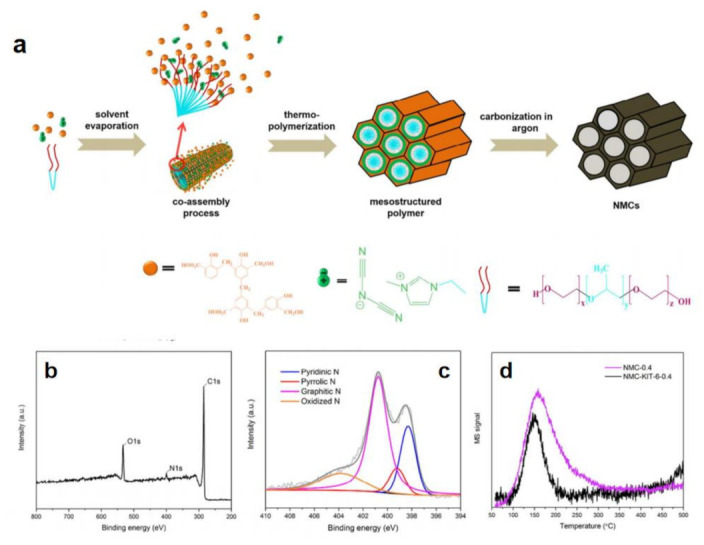
Schematic illustration of synthesis of IL-assisted porous nitrogen-rich carbon materials (**a**), their survey spectra (**b**) and N 1s (**c**), XPS spectra as well as CO_2_−TPD (**d**). Reprinted with permission from ref. [72], copyright 2017 ACS publications.

The higher heteroatom content can afford the higher positive charge density of C atom so that corresponding heteroatom-C catalysts show better efficiency to a certain extent [72]. However, it is difficult to obtain products with higher heteroatom content by relying solely on conventional ILs. It is necessary to design ILs with high heteroatom content. Taking nitrogen doped carbon as an example, cyano-based ILs are good candidates, such as 1-cyanomethyl-3-methylimidazolium ([MCNIM]^+^) cation, dicyanamide (DCA) anion, among others [72,77,78,79]. Cyano- can effectively increase nitrogen content of C nanomaterials. The Thomas group prepared porous nitrogen-rich carbon materials through using resol for forming the bulk mesoporous carbon structure and 1-ethyl-3-methylimidazolium dicyanamide ([EMIM]DCA) (Figure 3a–c) [72]. 

X-ray photoelectron spectroscopy (XPS) and temperature-programmed desorption of carbon dioxide (CO_2_-TPD) indicated that nitrogen atoms are successfully enriched on the pore surface (Figure 3d). The prepared catalysts exhibited excellent HER activity due to the synergies between N and the open mesoporous channel structure. In addition, other additives can also be further added into reaction system to increase the content of heteroatoms. Adding the compounds containing N, e.g., guanine, adenine, cytosine, uracil as well as thymine into cyanamide-based ILs can further increase N content to enhance catalytic activity [72].

### 3.2. Ionic Liquids as Reactants to Prepare Transition-Metal Electrocatalysts for Water Splitting

Among the electrolytic water catalysts, transition-metal catalysts involving Fe, Co, Ni are important research branch, mainly including metal elements, alloys, transition metal oxides, phosphides, chalcogenides, carbides, nitrides, etc. [80]. The catalysts need to have a large specific surface area, rich active site and short mass transfer distance because the electrocatalytic process mainly occurs on the surface of catalysts [81,82]. In addition, it is necessary to have a promising charge distribution to ensure excellent electronic transmission performance, so as to reduce overvoltage. Therefore, increasing the number of active sites and the intrinsic activity of a single active site is regulating strategies for catalytic performance [83]. These factors are closely related to the composition, structure and size of catalysts. The special properties and liquid structure of ILs provide different growth environment for catalysts from ordinary solvents. Therefore, ILs have the ability to tailor the morphologies and electronic structures of catalysts. The IL-mediated preparation strategy not only stabilize metal micro/nanomaterials, but also introduce heteroatoms into metal catalysts in situ. Therefore, ILs can subtly change the composition, structure and surface area of the catalysts, thus providing a good strategy for screening excellent catalysts. Therefore, IL-derived transition metal catalysts have attracted much attention in recent years.

#### 3.2.1. IL-Derived Single Transition Metal or Alloy

Single metal or alloy is widely used in the electrolysis of water [84,85,86]. Flower-like Cu_0.81_Ni_0.19_ alloy doped N, P, and F is obtained from Cu^2+^ and nickel foam in [BMIM]PF_6_ by employing convenient one-pot hydrothermal strategy, in which IL provides heteroatoms. This ingenious design simply obtains one of the most efficient copper-based alloy catalysts. This catalyst exhibits excellent property to HER and OER with overpotentials of 88 mV and 198 mV at 10 mA cm^−2^ in 1.0 M KOH, respectively. Obviously, the mediation of ILs simply obtain heteroatom-doped metal catalysts with favorable structures, synergistically promoting catalytic performance.

It has been known that the small particle sizes favor the exposure of the active sites, being conducive to the improvement of catalytic activity. However, the small size causes nano/micro-particles to aggregate easily, leading to their relatively small surface area and active sites. For this reason, it is necessary to select appropriate substrate materials. Carbon-based materials with a porous and large surface are a kind of good substrate materials. Integrating metals with them will hopefully make the metal active species evenly distribute on the carbon-based materials [87,88,89]. The synergistic effect between metal and carbon-based species is expected to increase the electron transport performance, being beneficial to the catalytic activity of micro/nanomaterials. 

As mentioned in Section 3.1, IL precursors can synthesize C-based materials. Therefore, ILs can derive metal-doped carbon materials by adding metal components to IL or poly(IL)s. IL-derived Co-based N-doped carbon (Co−N−C) materials are typical representatives of electrocatalysts. Co−N−C catalysts were obtained by mixing Co salt and IL precursor using pyrolysis technology. Wang and co-workers devised a clever method to obtain carbon nano-porous membranes using poly(IL)s [50]. They mixed anionic poly(acrylic acid) and cationic [PCMVIM]Tf_2_N to form a precursor with porous network through electrostatic crosslink. In general, the morphology of a precursor tends to collapse during the process of carbonization. In this system, however, the synergistic effect of the initially crosslinked structure and thermally stable network intermediates formed during carbonization process might keep the structure unchanged. Then, loading with Co nanoparticles on this N−C membrane obtained an active bifunctional electrocatalyst Co−N−C for both HER and OER. It showed high-performance HER activity (η_10_ = 158 mV) as well as remarkable OER activity (η_10_ = 199 mV).

**Figure 4:**
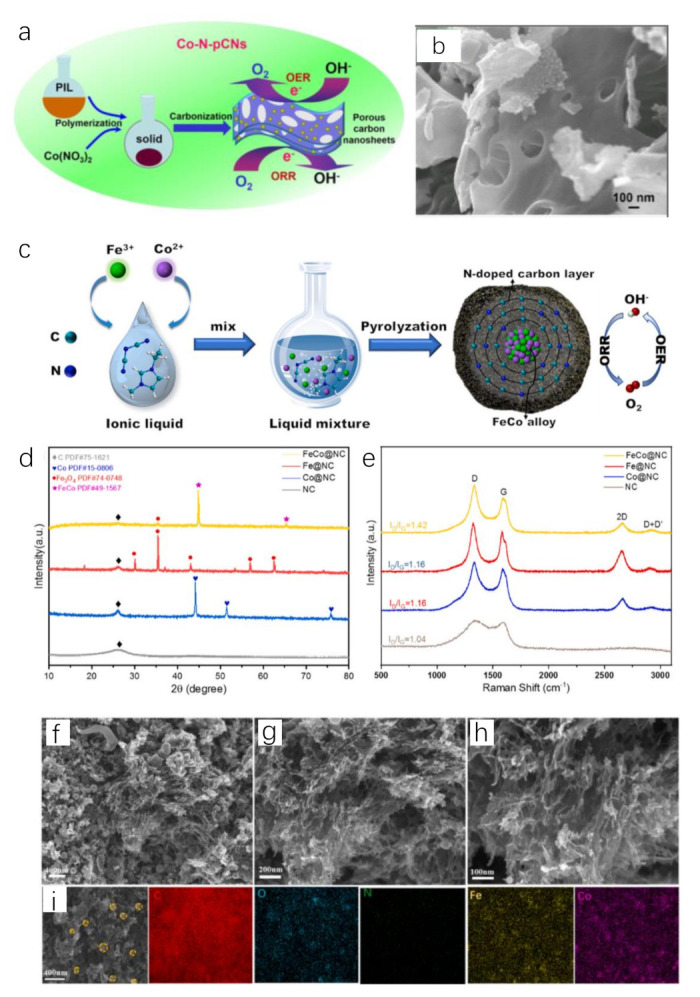
Schematic illustration for the fabrication process of 2D Co-N-C nanosheets (**a**), SEM image of 2D Co−N−C nanosheets (**b**). Reprinted with permission from ref. [91], copyright 2017 wiley. Schematic illustration for the fabrication process of FeCo@NC (**c**), XRD patterns (◊(gray), 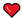
(blue), ●(red), ★(pink) on behalf of C, Co, Fe_3_O_4_, FeCo, respectively) (**d**) and Raman spectra (**e**) of FeCo@NC and control samples. SEM images (**f**–**h**) and EDS elemental mapping images (**i**) of FeCo@NC. Reprinted with permission from ref. [92], copyright 2022 Elsevier.

Poly(1-vinyl-3-methylimidazolium nitrate) (poly[HVIM]NO_3_) is an explosive poly(IL) because the thermal decomposition of NO_3_^−^ releases high concentration of nitrogen oxides [90]. Thus, it is a good pore-forming agent. Using this IL to prepare catalysts will effectively increase the catalytic area. Heating the mixture of cobalt pentazolate and poly[HVIM]NO_3_ can obtain porous structure in layers. Co atoms were homogeneously distributed on the layers of Co−N−C catalyst. Still using poly[HVIM]NO_3_, 2D Co−N−C nanosheets with porous structure are constructed by carbonizing the mixture of poly(IL) and Co(NO_3_)_2_ (Figure 4a,b) [91]. It exhibits a considerable overpotential of 400 mV at 10 mA cm^−2^ with Tafel slope of 127.4 mV dec^−1^ for OER.

The flexibility of M−N−C preparation inspires researchers to add additional element sources into the system of Co salt and ILs to obtain complex Co−N−C. Wang et al. prepared FeCo alloy encapsulated in N-doped carbon substrate using FeCl_3_, CoCl_2_ and [EMIM]DCA through annealing method (Figure 4c–i) [92]. IL was able to act as a solvent for metal ions while as N, C precursor with high carbon yield after pyrolysis. The solvation between metal ions and ILs improved the dispersion of metal ions and the uniformity of catalyst species after annealing. As-prepared FeCo@NC showed excellent OER and ORR performance. The overpotential was only 280 mV to reach 10 mA cm^−2^ for OER.

#### 3.2.2. IL-Derived Metal Sulfides

**Figure 5:**
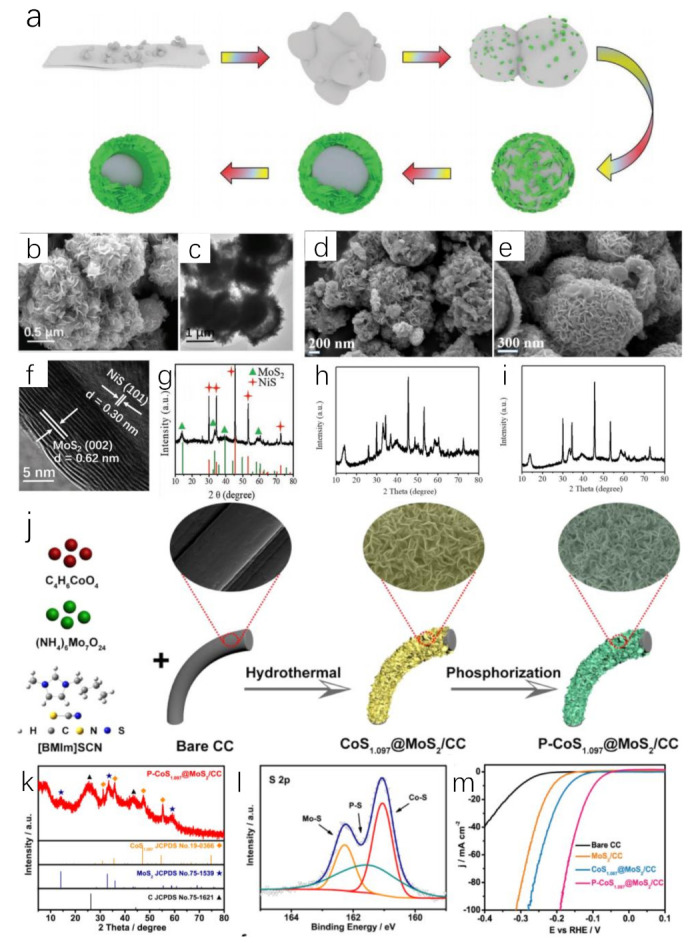
Schematic illustration of the formation mechanism of the yolk–shell nanostructure (**a**), SEM (**b**) and TEM (**c**) images of [BMIM]SCN−derived MoS_2_/NiS, SEM image of the KSCN−derived products (**d**), SEM image of the [BMIM]Cl/KSCN-derived products (**e**), HRTEM image (**f**) and XRD pattern (▲(green), ✦(red) represent MoS_2_ and NiS, respectively) (**g**) of [BMIM]SCN−derived MoS_2_/NiS, XRD pattern of KSCN−derived products (**h**), XRD pattern of [BMIM]Cl/KSCN−derived products (**i**). Reprinted with permission from ref. [93], copyright 2019 wiley. Schematic illustration of the synthetic procedure of P−CoS_1.097_@MoS_2_ nanosheets (**j**), XRD pattern (◊(orange), ★(purple), ▲(black) represent CoS_1.097_, MoS_2_, C, respectively) (**k**) and S 2p XRS spectra (**l**) of P−CoS_1.097_@MoS_2_ nanosheets, LSV curves of P−CoS_1.097_@MoS_2_ and control samples for HER (**m**). Reprinted with permission from ref. [95], copyright 2021 Elsevier.

Metal sulfides are ideal electrocatalysts for water splitting. The addition of S will optimize the structure of transition metal catalysts and expose more active sites. ILs containing S element, such as [BMIM]SCN, have been found to be smart S sources to replace thiourea, KSCN or other S sources in the preparation of metal sulfides [93,94]. Adding Ni or Co salt to the system of [BMIM]SCN and (NH_4_)_6_Mo_7_O_24_·4H_2_O will obtain IL-derived MoS_2_/NiS hybrid (Figure 5a–i) [93] or CoS_1.097_@MoS_2_ via hydrothermal method (Figure 5j–m) [95]. For MoS_2_/NiS, [BMIM]SCN played important roles during the synthesis process. Firstly, the intrinsic properties of ILs change the polarity of the synthesis system, while they can affect the solubility of the compounds because of the low surface tension and good thermal stability. This allowed the metal compound to dissolve and participate in the reaction. Secondly, [BMIM]SCN as reactant followed a novel reaction path. [BMIM]SCN as a novel S source preferentially reacted with Ni^2+^ to form nickel sulfide, while [BMIM]SCN reacted with (NH_4_)_6_Mo_7_O_24_·4H_2_O to generate [BMIM]_2_Mo_4_O_13_. The latter gradually dissolved while adsorbed on the surface of nickel sulfide, resulting in the formation of MoS_2_ crystal nucleus. Thirdly, [BMIM]SCN acted as a template to modulate the well-defined yolk−shell nanostructure (Figure 5b,c,f,g). This effect could be confirmed via control experiments [93]. Using equimolar KSCN to replace [BMIM]SCN, some nanoplates coexisted with a significant number of nanoparticles. This phenomenon showed that KSCN had no structure-orienting function (Figure 5d,h). However, adding [BMIM]Cl into the reaction system containing KSCN led to the appearance of well-defined yolk–shell nanostructure (Figure 5e,i). These experiments verified the template effect of [BMIM]SCN. This effect stemmed from the strong ionic nature of [BMIM]SCN. IL could form the intense interaction with nanoparticles, promoting the synthesis of well-defined layers. With the hollow structure and favorable interface effect, IL-derived MoS_2_/NiS hybrid microspheres presented HER (η_10_ = 244 mV) and OER activity (η_10_ = 350 mV) in an alkaline aqueous solution. When it was applied for catalyzing overall water splitting, an output voltage of 1.64 V at 10 mA cm^−2^ was required in the same electrolyte, being lower than that of Pt/C−IrO_2_ electrolysis cells (1.70 V) [93]. The role of [BMIM]SCN was further studied during the preparation of CoS_1.097_@MoS_2_/CC heterostructure [95]. The H atom at C2 position in [BMIM]^+^ has Lewis acidity. It can be easily adsorbed on the negative charge center S^2−^ of CoS_1.097_ and MoS_2_ through electrostatic interaction and hydrogen bond, preventing their aggregation. Meanwhile, CoS_1.097_@MoS_2_/CC can also reduce aggregation from the steric hindrance effect of [BMIM]^+^. It was also beneficial to the adequate exposure of electrocatalytic active sites and rapid electron transport. Further phosphating IL-derived metal sulfide, a novel P-CoS_1.097_@MoS_2_ nanosheets was synthesized [95]. The prepared P-CoS_1.097_@MoS_2_ nanosheets had a low overpotential in both acidic and alkaline solutions, which required overpotential of 98 mV and 88 mV to reach 10 mA cm^−2^, respectively.

#### 3.2.3. IL-Derived Metal Phosphides or Phosphates

In phosphides, the phosphorus atom increases the distance between the metal atoms, weakens the interaction between the atoms to a certain extent and the d orbital shrinks, thus changing the density of energy states of the Fermi energy level. Therefore, transition metal phosphides have characteristics similar to noble metals, which are called “quasi Pt catalysts” for water electrolysis [96]. The traditional P sources mainly include phosphorus, PH_3_, NaH_2_PO_2_ and tributylphosphine (TBP), trioctylphosphine (TOP), etc. However, most of them are toxic and flammable. Therefore, exploring safe and green P sources has become a matter of serious concern [97]. Owing to the designability of IL, both quaternary phosphonium salt and PF_4_^−^-based ILs can be used as phosphorus sources.

**Figure 6:**
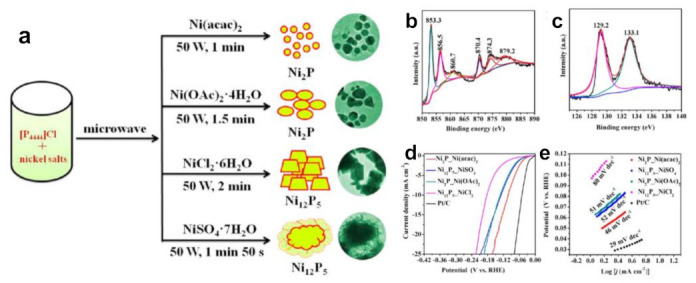
Typical schematic illustration for synthesizing various nickel phosphides controlled by the counter anions of nickel salts (**a**), XPS spectra of the Ni 2p (**b**) and P 2p (**c**) regions for Ni_2_P_Ni(acac)_2_ nanocrystals, polarization curves of the as-synthesized Ni_x_P_y_ catalysts (**d**) and Tafel plots of catalysts (**e**) for HER. Reprinted with permission from ref. [24], copyright 2018 ACS.

Our group firstly used tetrabutylphosphonium chloride ([P_4444_]Cl) to prepare Ni_x_P_y_ nanomaterials using a microwave-driven approach (Figure 6a–c) [24]. The size of as-obtained nickel phosphide particles was small and uniform under the synergistic effect of microwave and IL. The average particle size of Ni_2_P nanoparticles prepared by Ni(acac)_2_ was only 12 ± 3.3 nm. We speculated that the reason was that ILs were adsorbed on the surface of nanoparticles, causing them to repel each other and prevent aggregation. Moreover, changing the counter anions of Ni salts could easily adjust the nickel phosphides with different phases. Ni(acac)_2_ as well as Ni(Oac)_2_·4H_2_O offered Ni_2_P nanocrystals, whereas NiSO_4_·7H_2_O and NiCl_2_·6H_2_O yielded Ni_12_P_5_ nanomaterials. The reason for formation of different crystal phases was related to the interaction force between anions and cations in different nickel salts. All as-prepared phosphides were pure phases. Microwave heating is fast and efficient, avoiding the limitations of calcination in a tube furnace involving time-consuming and tedious operation. Their electrocatalytic behavior of Ni_x_P_y_ toward HER in an acidic medium was investigated. The negatively charged P atoms enrich and accept protons, while the metal centers act as electron collectors. P alloyed into Ni can lower the energy barrier for H adsorption and tune the electronic structure. P and Ni synergistically boost the intrinsic activity for HER. It was found that the as-obtained Ni_2_P nanomaterials showed better elecrocatalytic efficiency than Ni_12_P_5_ for HER (Figure 6d,e). Ni_2_P nanomaterials from Ni(acac)_2_ required a small overpotential of 102 mV to achieve a current density of 10 mA cm^−2^ with a Tafel slope of 46 mV dec^−1^. These experimental results fully proved that phosphonium-based ILs were not only novel P sources but also good reaction media, templates and stabilizers, and could obtain different pure phase of nickel phosphides with different nickel salts. Obviously, the mediation of phosphonium-based ILs to nickel phosphides has the ability to modulate the catalytic activity. This achievement has aroused widespread concern on phosphonium salts as P sources to synthesize other phosphides successively [98,99,100]. 

ILs containing transition metal anions (such as FeCl_4_^−^, NiCl_3_^−^, and CoCl_3_^−^) have been extensively studied [101,102,103,104]. This kind of ILs has the ability to provide metal elements for catalytic materials for water splitting [105,106]. A more ingenious design is to use metal anions and quaternary phosphonium salt cations to form ILs, such as [P_66614_]_2_CoCl_4_ [98,99], [P(C_6_H_13_)_3_C_14_H_29_]FeCl_4_ [107], among others. These ILs are directly used as reactants to obtain metal phosphides through the reaction between their own anions and cations using [P_66614_]_2_CoCl_4_ as phosphorus and metal dual-source fabricated Co_2_P/CNTs via one-step phosphidation, in which CNTs enhanced the electrical conductivity and contributed to the formation of Co_2_P [98,99]. In acidic solution, Co_2_P/CNTs showed an enhanced HER catalytic activity which had onset overpotential of 80 mV as well as Tafel slope of 58 mV dec^−1^, while arrived current densities of 10 mA cm^−2^ and 20 mA cm^−2^ at overpotentials of 135 mV and 170 mV, respectively. Similarly, Fe_2_P was formed from [P(C_6_H_13_)_3_C_14_H_29_]FeCl_4_ in situ on the CNTs [107]. Fe_2_P/CNT exhibited excellent HER performance with current densities of 10 and 20 mA cm^−2^ at overpotentials of 115 and 150 mV, respectively. FeP(MBMG)/CNT, stemmed from [MBMG]FeCl_3_Br with CNTs and NaH_2_PO_2_ [105], exhibited high activity for water splitting. This strategy can make not only the phosphorus and metal elements arrange according to the IL-induced structure but also evenly distribute and have many active sites. In addition, using both cation and anion of IL to participate in the reaction can reduce side reactions and improve reproducibility. Recently, Fe_7_(PO_4_)_6_ was transformed from tetrabutylphosphoniumtetrachloroferrate ([P(C_4_H_9_)_4_]FeCl_4_) or trihexyl(tetradecyl)phosphonium tetrachloroferrate ([P(C_6_H_13_)_3_C_14_H_29_]FeCl_4_). It exhibited an onset overpotential of 120 mV with Tafel slope of 32.9 mV dec^−1^ for HER, while there was an onset potential of 1.48 V with Tafel slope of 73.3 mV dec^−1^ for OER [108]. 

Our group explored the application of other new IL containing phosphorus to synthesize phosphorus-contained catalysts. We reacted octylamine and hypophosphorous acid to design a proton-type IL, octylamine/hypophosphorous acid [109]. NiCl_2_ was dissolved in this proton-type IL. Ni_2_P_4_O_12_ with good crystallinity was obtained through calcining at high temperature. The prepared Ni_2_P_4_O_12_ had good hydrogen evolution performance. Under alkaline conditions, the overpotential required at a current density of 10 mA cm^−2^ was only 116 mV, while the Tafel slope was only 97 mV dec^−1^. Compared with the traditional phosphorus sources, the proton-type IL is cheap and easy to obtain, which is conducive to large-scale production.

PF_6_-based ILs can also be used as phosphorus sources for metal phosphides. Using metal compound solution and [BMIM]PF_6_ as ink, metal phosphides (MoP, CoP, NiP, FeP) wrapped by carbon fiber are obtained with simple inkjet printing technology [110]. [BMIM]PF_6_ acts as a source of phosphorus and carbon through its own decomposition. The prepared catalyst has good electrolytic water hydrogen evolution performance in acidity, alkalinity and neutrality. 

In the system of Co(NO_3_)_2_·6H_2_O and biomass-based protic ionic liquids (BILs), adding phosphoric acid causes the synthesis of porous carbon-coated CoP nanocrystals through a simple one-pot carbonization process [111]. In the process of pursuing green chemistry, BILs, as green solvents with inherent properties, gradually transforms into N, P co-doped porous carbon (NPC). The synergistic effect between CoP nanocrystals and NPC is helpful to improve the electron transfer ability and prevent the accumulation of cobalt phosphide. CoP@NPC-900 obtained by calcining at 900 °C presents the superiorly catalytic performance for HER. In acid solutions, it has low overpotential (181 mV) at 10 mA cm^−2^ and small Tafel slope (59 mV dec^−1^). Density functional theory (DFT) calculation further proves that the material has excellent HER catalytic performance.

#### 3.2.4. IL-Derived Metal Carbides

**Figure 7:**
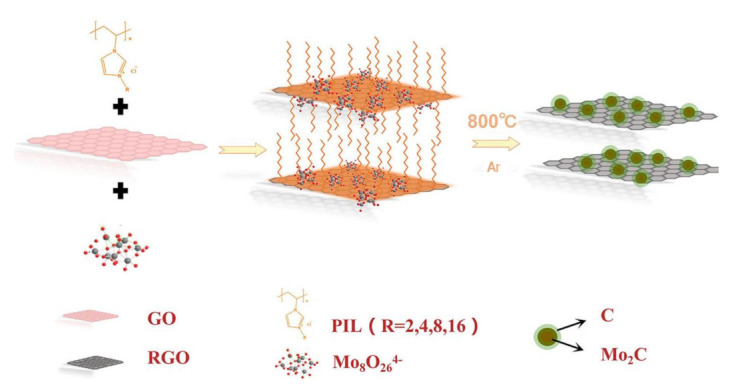
Schematic illustration of the synthetic process of Mo_2_C−RGO. Reprinted with permission from ref. [113], copyright 2019 ACS publication.

Metal carbides have also been widely investigated as highly active electrocatalysts in water splitting. However, they are difficult to prepare using conventional methods. Moreover, metal carbide nanoparticles are easily sintered and aggregated during carbonization at high temperature. The intervention of Ils solves these problems well. [BMIM]_2_MoO_4_-bearing MoO_4_^2−^ anion was smartly designed [112]. It would be a promising precursor to synthesize MoC because it could offer simultaneously both C and Mo atoms. In order to obtain an ordered structure, [BMIM]_2_MoO_4_ was preferentially primed into mesoporous SBA−15 silica as hard template. Then, the ordered mesoporous MoC nanoparticles were successfully formed through one-step pyrolysis at optimized temperature. Linked with graphite carbons, IL-derived MoC@C with an ordered mesoporous structure was fabricated, in which [BMIM]_2_MoO_4_ acted as the only precursor for the reaction of carbon and Mo. It was an efficient electrocatalyst for HER. However, the sublimation of Mo species and strong interaction with template were harmful to the controllability of structure. In addition, this nano-casting synthesis limits the volume change of metal species during pyrolysis process. Recently, poly(IL) was used as a bridge between the olyoxometalate and reduced graphene oxide (RGO) to evenly fix the Mo species on the RGO surface (Figure 7) [113]. Annealing the mixture of (NH_4_)_6_Mo_7_O_24_·4H_2_O, imidazolium-based poly(IL) and RGO obtained Mo_2_C-RGO. Poly(IL) could interact with graphene through π-π interaction or cation-π stacking, effectively resisting sintering and agglomeration of Mo_2_C nanoparticles at high temperature. Therefore, poly(IL) was not only a carbon source but also a good nanoparticle dispersant in this process. The mediation of poly(IL) avoided the disadvantages of hard templates to a certain extent. Mo_2_C−RGO exhibited excellent activity for HER in 0.5 M H_2_SO_4_ with a low overpotential (99 mV to obtain 10 mA cm^−2^) and a small Tafel slope of 54.6 mV dec^−1^. No obvious degradation was noticed after 20 h of continuous catalysis.

#### 3.2.5. IL-Derived Metal Nitrides

In recent years, transition metal nitrides (TMNs) have attracted much attention due to their unique electronic properties and long-term stability. On the one hand, the addition of N atoms changes the properties of d in the band the parent metal, increasing the d electron density in TMNs while leading to the shrinkage of metal d belt [114]. Therefore, the electronic structure of TMNs is more similar to that of noble metals (such as Pd and Pt), thus showing strong HER activity. On the other hand, N atom can be nested in the lattice gap due to its small atomic radius; therefore, the arrangement of metal atoms always keeps close packing, which endows TMNs with high electronic conductivity. These promising properties, combined with high corrosion resistance, make TMNs more reliable than metals or metal alloys.

Compared with metal sulfides and phosphides, transition metal nitrides are relatively difficult to prepare. Applying Ils to prepare transition metal nitrides has been studied, and has been applied to overall water splitting [115,116]. The Zhang and Wang groups have studied the synthesis of IL-derived heteroatom-doped metal nitride catalysts [117,118,119]. They injected the appropriate amount of [BMIM]PF_6_ into a solution containing Ni^2+^: Co^2+^ (mass ratio of 1:1) [117]. Nanoroded P,F-co-doped Ni_1.5_Co_1.5_N hybrids were synthesized via solvothermal treatment, which N, P and F atoms were offered by [BMIM]PF_6_. In 1.0 M KOH, it required overpotential of 280 mV at 10 mA cm^−2^ with the Tafel slope of 66.1 mV dec^−1^ (Table 1).

## 4. Summary and Outlook

The great progress of Ils in the preparation of electrocatalysts for water splitting has been achieved. Ils can participate in reactions as reactants (modifiers or reactive reagents) besides media and templates. As modifiers, they have the ability to change surface properties and electronic structures of catalysts, which are conducive to catalytic performance. As reactants, they are safe and efficient to directly participate in the synthesis of catalysts. Moreover, Ils have the opportunity to regulate the growth environment and growth mechanisms to control the morphology and size of nanomaterials through their supramolecular structure. Applied Ils have made great achievements in the morphology, properties and green synthesis of catalysts. They have opened up a meaningful way for the preparation and efficiency improvement of electrocatalysts. However, the studies on electrocatalysts based on Ils still have some scientific problems to be solved. 

### 4.1. Problem about Theoretical Guidance and Controllable Preparation of Electrocatalysts in Ionic Liquids 

At present, the preparation of electrocatalysts derived from Ils has some contingency in structure and properties. Researchers intend to explore this problem from different perspectives [120,121]. Li et al. deeply discussed the relationship between Ils and crystal nucleation kinetics, proving that Ils can affect crystal nucleation [122,123]. Wegner et al. tried to summarize the research on the interdependence between Ils and metal nanoparticles [124]. However, the mechanism stemmed from Ils for crystal morphology is still unclear, while the reaction mass transfer and the crystallization stage of nanomaterials was not discussed. To date, only ambiguous rules have been obtained, such as about how the size of nanoparticles increases with the increase in the anion molecular volume of Ils [125]. It is necessary to understand the interaction between nanoparticles and Ils in detail to design more reasonable nanomaterials.

### 4.2. Problem about the Post-Treatment for Ionic Liquids

Ils are regarded as green solvents due to their low vapor pressure and non-volatile characteristics. However, the toxicity of Ils was discussed at the first Green Solvent Catalysis meeting in 2002. Subsequently, there have been an increasing number of studies on the toxicity of Ils [126]. The toxicity of Ils has prompted imminent research on post-treatment. At present, the recovery methods of Ils mainly include vacuum distillation, membrane separation, salting out, and extraction. However, these methods have disadvantages such as high resource consumption or only target specific Ils without universality, which cannot be fully extended to industrial production. How to dispose of waste Ils has become a world problem. From this review, we can see that using Ils as active reagents is an effective means to reduce IL emissions. In particular, the simultaneous conversion of cation and anion of IL into catalyst components will be a development direction in the field of catalysis.

### 4.3. Research on More Environmentally Friendly Alternatives

With the in-depth and extensive study of Ils, several problems that perplex researchers have been found, such as their high price and difficulty in purification. Therefore, deep eutectic solvents (DESs) have attracted people’s attention [127,128]. DESs are formed by hydrogen bond acceptors (HBAs) and hydrogen bond donors (HBDs) through hydrogen bond at a certain molar ratio, and have a relatively low freezing point. DESs have properties similar to Ils, such as a low freezing point, high solubility, good conductivity, excellent thermal stability and high viscosity. DESs have been studied in the field of catalyst preparation. It is found that DESs can prepare electrolytic water catalysts with special high performance, similar to IL-derived catalysts. Importantly, DESs have more advantages than Ils, such as convenient purification, low price, good biodegradability and biocompatibility. The design of DESs to prepare catalysts is more flexible and the operation is simpler than that of Ils. Therefore, DESs are more promising than Ils in industry, which can be used as substitutes for Ils in the field of catalyst preparation.

## Figures and Tables

**Table 1:** Summary of HER, OER and overall water splitting performance of IL-derived catalysts involved in the Section 3.

Catalyst	Applied IL	The Role of IL	Preparation on Method	Catalytic Performance								Ref.
				HER			OER			Overall Water Splitting		
				Electrolyte	ƞ (mV)@ Current Density (mA cm^−2^)	Tafel Slope (mV dec^−1^)	Electrolyte	ƞ (mV)@ Current Density (mA cm^−2^)	Tafel Slope (mV dec^−1^)	Electrolyte	Potential (V)@Current Density (mA cm^−2^)	
NiP_2_	[P_4444_]Cl	As a solvent, template and reactant (providing P)	microwave	0.5 M H_2_SO_4_	102@10	46						[24]
CNT−IM−Cl	[BMIM]Cl	As a modifier	reflux	0.5 M H_2_SO_4_	135@10	38						[41]
CoW_12_−IL−GO	imidazolium Ils	As a modifier; Regulating catalytic activity	reflux				0.1 M Na_2_SO_4_	269@10	92			[48]
Co−N−C	interpolyelectrolyte complexation between [PCMVIM]Tf_2_N and PAA	As a modifier	direct pyrolysis	1 M KOH	158@10	93.4	1 M KOH	199@10	66.8			[50]
imidazolium-based poly(IL)	poly(VIMM)	As a catalyst	surface initiated atom transfer radical polymerization						0.1 M KOH			[52]
Pt/C/poly(IL)	poly(VIMM)	As a modifier	surface initiated atom transfer radical polymerization						0.1 M KOH			[52]
GC/Poly(IL)/Pd	PAMI	As a modifier	surface initiated atom transfer radical polymerization	0.5 M H_2_SO_4_	170@10	83						[55]
Co−poly(IL)/CNT	SSIL	As a modifier and dispersant	assembly				1 M KOH	430@10	41.6			[56]
NiS_2_−MoS_2_/PVEIB/Ppy/GO	PVEIB	As a modifier	surface initiated atom transfer radical polymerization	0.5 M H_2_SO_4_	45@10	32.5						[59]
P,N,F−rGO	[BMIM]PF_6_	As a reagent (providing P, N, F and C)	carbonization				1 M KOH	170@176	73			[69]
BNF−rGO	[BMIM]BF_4_	As a reagent (providing B, N, F and C)	carbonization				1 M KOH	110@236	90			[69]
N,S−graphene	[NMP]HSO_4_	As a reagent (providing N, S and C)	carbonization				0.1 M KOH	310@10	65			[70]
N−C	[EMIM]DCA	As a reagent (providing N and C)	evaporation-induced self-assembly method	0.5 M H_2_SO_4_	384.7@10	134						[72]
N,P−graphene	[MBMG]PF_6_	As a reagent (providing N, P and C)	carbonization	0.5 M H_2_SO_4_	210	88						[75]
N,S,P−C	[PCMVIM]Tf_2_N	As a reagent (providing N, S, P and C)	carbonization	0.5 M H_2_SO_4_	172	44.3	0.1 M KOH	460@10	88			[76]
N,S−C	Cyano-based Ils	As a reagent (providing N, S and C)	carbonization				0.1 M KOH	450@10	216			[79]
PIL−Ru/C	[MBVIM]Br	As a reagent (providing N and C) and dispersant	polymerize	1 M KOH	16@10	42						[89]
Co−N−C	Poly[HVIM]NO_3_	As a reagent (providing N and C) and dispersant	carbonization				1 M KOH	400@10	127.4			[91]
Fe, Co−N−C	[EMIM]DCA	As a solvent and reagent (providing N and C)	pyrolysis				1 M KOH	280@10	153			[92]
MoS_2_/NiS	[BMIM]SCN	As a solvent, templet and reagent (providing S); Changing the reaction path	hydrothermal method	1 M KOH	244@10	97	1 M KOH	350@10	108			[93]
P−CoS_1.097_@MoS_2_	[BMIM]SCN	As solvent, templet and reagent (providing S); Changing the reaction path	hydrothermal reaction	0.5 M H_2_SO_4_	98@10	51						[95]
P−CoS_1.097_@MoS_2_	[BMIM]SCN	As a solvent, templet and reagent (providing S); Changing the reaction path	hydrothermal reaction	1 M KOH	88@10	74.4						[95]
Co_2_P/CNTs	[P_66614_]_2_CoCl_4_	As a solventand reagent (providing Co and P)	microwave method	0.5 M H_2_SO_4_	135@10	58						[98]
Co_2_P/CNTs	[P_66614_]_2_CoCl_4_	As a solvent and reagent (providing Co and P)	annealing method	0.5 M H_2_SO_4_	150@10	47						[99]
CNT−supported iron phosphating	[MBMG]FeCl_3_Br	As a reagent (providing Fe)	heated in an inert atmosphere	0.5 M H_2_SO_4_	155@10	75.9						[105]
CuCo_2_S_4_	[BuPy]CoCl_4_	As a reagent (providing Co)	hot-injection method				1 M KOH	230@10	211			[106]
Fe_2_P/CNTs	[P(C_6_H_13_)_3_C_14_H_29_]FeCl_4_	As a solvent and reagent (providing Fe and P)	annealing method	0.5 M H_2_SO_4_	115@10	68						[107]
Fe_7_(PO_4_)_6_	[P(C_4_H_9_)_4_] FeCl_4_	As a solvent and reagent (providing Fe and P)	microwave radiation	0.5 M H_2_SO_4_		32.9	1 M KOH		73.3			[108]
Ni_2_P_4_O_12_	Octylamine/hypophosphorous	As a solvent and reagent (providing P)	annealing method	0.5 M H_2_SO_4_	116@10	97						[109]
MoP wrapped by carbon fiber	[BMIM]PF_6_	As a reagent (providing P)	inkjet printing technology	0.5 M H_2_SO_4_	87@10	49.1						[110]
MoC	[BMIM]_2_MoO_4_	As a reagent (providing Mo and C)	annealing method	0.5 M H_2_SO_4_	110@10							[112]
Mo_2_C−RGO	imidazolium Ils	As a reagent (providing C) and dispersant	annealing method	0.5 M H_2_SO_4_	99@10	54.6						[113]
P,F−Ni_1.5_Co_1.5_N	[BMIM]PF_6_	As a solvent and reagent (providing P, F and N)	solvothermal method				1 M KOH	280@10	66.1			[117]
Cu_0.81_Ni_0.19_	[BMIM]PF_6_	As a solvent	a hydrothermal treatment	1 M KOH	88@10	91	1 M KOH	198@10	76	1 M KOH	1.58@10	[119]

## Data Availability

Not applicable.
